# A novel *TBK1* mutation in a family with diverse frontotemporal dementia spectrum disorders

**DOI:** 10.1101/mcs.a003913

**Published:** 2019-06

**Authors:** Ruth Lamb, Jonathan D. Rohrer, Raquel Real, Steven J. Lubbe, Adrian J. Waite, Derek J. Blake, R. Jon Walters, Tammaryn Lashley, Tamas Revesz, Janice L. Holton, Huw R. Morris

**Affiliations:** 1 Department of Clinical and Movement Neurosciences, Dementia Research Centre, UCL Queen Square Institute of Neurology, London WC1N 3BG, United Kingdom;; 2 Department of Neurodegenerative Disease, Dementia Research Centre, UCL Queen Square Institute of Neurology, London WC1N 3BG, United Kingdom;; 3 Ken and Ruth Davee Department of Neurology, Feinberg School of Medicine, Northwestern University, Chicago, Illinois 60611, USA;; 4 Division of Psychological Medicine and Clinical Neurosciences, MRC Centre for Neuropsychiatric Genetics and Genomics, School of Medicine, Cardiff University, Cardiff CF24 4HQ, United Kingdom;; 5 Department of Neurology, Morriston Hospital, Swansea SA6 6NL, United Kingdom;; 6 Queen Square Brain Bank for Neurological Disorders, UCL Queen Square Institute of Neurology, London WC1N 1PJ, United Kingdom

**Keywords:** progressive extrapyramidal movement disorder

## Abstract

Mutations in the TANK-binding kinase 1 (*TBK1*) gene have recently been shown to cause frontotemporal dementia (FTD) and amyotrophic lateral sclerosis (ALS). The phenotype is highly variable and has been associated with behavioral variant FTD, primary progressive aphasia, and pure ALS. We describe the clinical, anatomical, and pathological features of a patient who developed corticobasal syndrome (CBS)/progressive nonfluent aphasia (PNFA) overlap. The patient presented with progressive speech difficulties and later developed an asymmetric akinetic–rigid syndrome. Neuroimaging showed asymmetrical frontal atrophy, predominantly affecting the right side. There was a strong family history of neurodegenerative disease with four out of seven siblings developing either dementia or ALS in their 50s and 60s. The patient died at the age of 71 and the brain was donated for postmortem analysis. Histopathological examination showed frontotemporal lobar degeneration TDP-43 type A pathology. Genetic screening did not reveal a mutation in the *GRN*, *MAPT*, or *C9orf72* genes, but exome sequencing revealed a novel p.E703X mutation in the *TBK1* gene. Although segregation data were not available, this loss-of-function mutation is highly likely to be pathogenic because it is predicted to disrupt TBK1/optineurin interaction and impair cellular autophagy. In conclusion, we show that *TBK1* mutations can be a cause of an atypical parkinsonian syndrome and screening should be considered in CBS patients with a family history of dementia or ALS.

## INTRODUCTION

Mutations in the tumor necrosis factor receptor-associated factor NF-κB activator (TANK)-binding kinase 1 (*TBK1*) gene, located on Chromosome 12, have recently been described as a cause of familial frontotemporal dementia (FTD) and amyotrophic lateral sclerosis (ALS) (Cirulli et al. 2015; Freischmidt et al. 2015; Pottier et al. 2015). Patients have usually presented with either behavioral variant FTD, pure ALS, or a combination of both (FTD–ALS), and multiple different syndromes have been observed within the same family (Van Mossevelde et al. 2016).

TBK1 is a 729-amino acid protein kinase that acts as a mediator of inflammation and autophagy (Marion 2014; Ahmad et al. 2016). It is a ubiquitously expressed serine–threonine kinase belonging to the “noncanonical IκB kinases” (IKKs) (Marion 2014; Ahmad et al. 2016). More than 40 mutations have been found in the *TBK1* gene that cause either a frameshift or a premature stop codon, likely leading to a truncated or absent protein product (Freischmidt et al. 2017). Similar to mutations in the progranulin (*GRN*) gene, these are likely to be pathogenic through haploinsufficiency (Freischmidt et al. 2015). More than 50 missense mutations have also been described with unclear pathogenicity (Freischmidt et al. 2017). Further functional work will be required to assess whether these are truly pathogenic or could represent risk factors for FTD and ALS.

Corticobasal syndrome (CBS) is a rare neurodegenerative disease characterized by progressive asymmetrical rigidity and apraxia (Armstrong et al. 2013). Other features include myoclonus, akinesia, dystonia, alien limb phenomena, and cognitive impairment. CBS is pathologically heterogeneous with only around half of cases being associated with the typical histological findings of corticobasal degeneration (Ling et al. 2010). Other cases are caused by progressive supranuclear palsy, Alzheimer's disease, and, more rarely, TDP-43 pathology. The majority of cases of CBS are sporadic but familial cases have been rarely described in association with mutations in *GRN*, microtubule-associated protein tau (*MAPT*), Chromosome 9 open reading frame 72 (*C9orf72*), and colony-stimulating factor 1 receptor (*CSF1R*) (Masellis et al. 2006; Rademakers et al. 2012; Anor et al. 2015).

In this report, we describe the clinical, radiological, and pathological features of a 59-yr-old woman who developed CBS in association with primary progressive aphasia caused by a novel *TBK1* mutation.

## RESULTS

### Clinical Presentation and Family History

**Figure 1.**
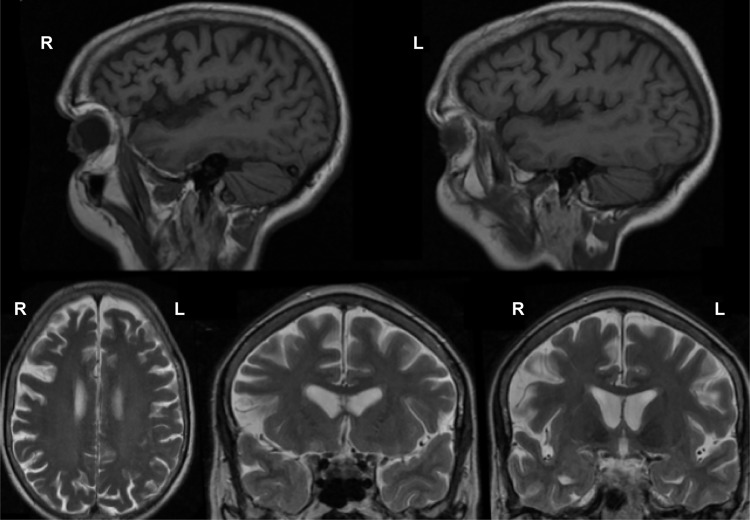
Magnetic resonance imaging scans demonstrating frontal lobe atrophy, with the right side more markedly affected. (*Top*) T1-weighted sagittal views. (*Bottom*) T2-weighted axial (*left*) and coronal (*middle* and *right*) views.

A right-handed woman presented at the age of 59 with a 6-mo history of progressive speech problems. Her initial symptoms were of knowing what she wanted to say but not being able to get the words out. There were no other cognitive, behavioral, or motor symptoms. Her symptoms slowly progressed over the next 2 yr and when assessed at the age of 62, her problems continued to be of impaired speech output without any other cognitive symptoms. Cranial nerve and limb neurological examinations were normal, but she had a reduced score of 81/100 on the Addenbrooke's Cognitive Examination (subscores: 18/18 attention and orientation, 20/26 memory, 7/14 fluency, 24/26 language, and 12/16 visuospatial). Formal psychometric testing was not performed but she was assessed on the Boston Naming Test, where she scored within the normal range (57/60). Blood tests and CSF examination were normal, as was electromyography. However, an MRI brain scan was abnormal, showing focal right frontal lobe atrophy (Fig. 1; Supplemental Movie S1). A diagnosis of PNFA was made.

**Figure 2.**
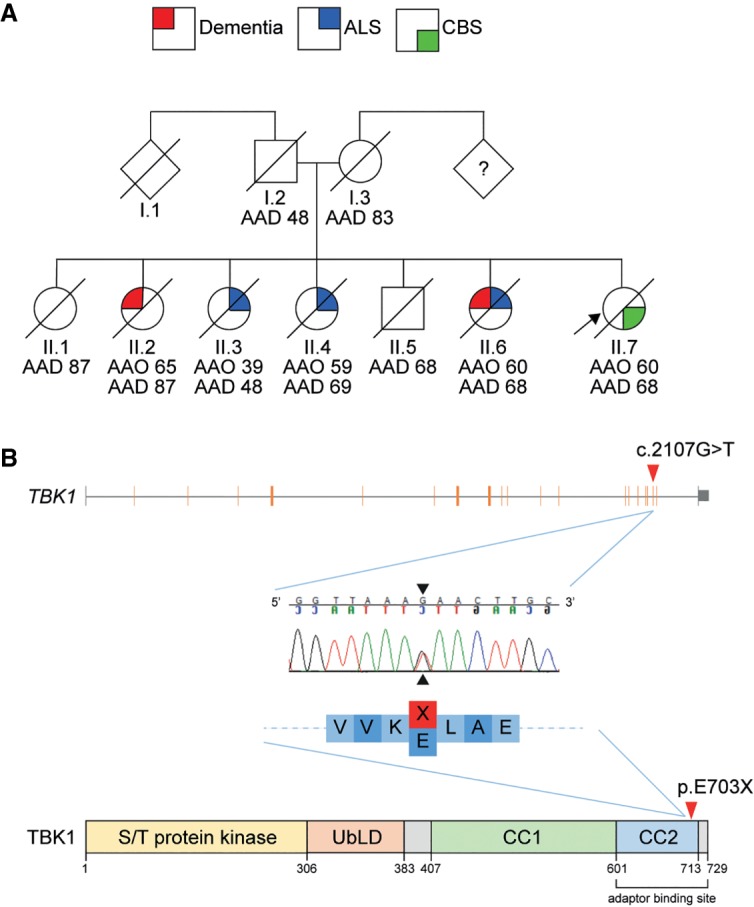
(*A*) Pedigree indicating the diversity of familial neurological disease present among family members. (*B*) Structure of the *TBK1* gene (NM_013254) and reserve-complement chromatogram showing the position of the c.2107G>T variant identified in the index case (II.7), leading to a 27-amino acid deletion in the CC2 domain of the TBK1 protein. (AAD) Age at death, (AAO) age at onset, (S/T) serine-threonine, (UbLD) ubiquitin-like domain, (CC1) coiled-coil domain 1, (CC2) coiled-coil domain 2.

Neither of her parents had a neurodegenerative illness, but her father died of myocardial infarction in his 40s. The mother died aged 83 of a stroke. She was one of seven siblings: One sister developed a rapidly progressive dementia in her 60s (II.2), two other sisters were diagnosed with ALS in their 40s (II.3) and 60s (II.4), and another sister was thought to have ALS and dementia with onset in her 50s (II.6), highlighting the variability of clinical presentations within the same family (Fig. 2A). II.2 first developed symptoms aged 65, presenting with a change in behavior. She became childlike in her demeanor and exhibited changes in appetite, developing a very sweet tooth and tending to overfill her mouth. Her behavior decline was rapid, deteriorating over a period of 6 mo. She was diagnosed with dementia and died aged 68. II.3 first developed symptoms aged 39 and was reported to have difficulties with her speech and swallowing, which progressed rapidly over a period of 3 mo; she was not reported to have any cognitive or behavioral difficulties. She was diagnosed with ALS and died aged 48. II.4 developed symptoms aged 59 and was diagnosed with ALS; however, she was not reported to have any difficulties with her speech or swallowing. Her symptoms progressed over a period of 12 mo. She died aged 69. II.6 developed symptoms aged 60. She was thought to have ALS and memory problems, but little was known of her condition as she reportedly became a recluse, refusing to leave the house or let anyone visit her. She died aged 68.

The patient's speech problems progressed and, when reassessed 4 yr into her illness, she had minimal speech output with evidence of orofacial apraxia. She had developed impaired swallowing and had had a PEG tube inserted. She had also developed an asymmetric akinetic–rigid syndrome with apraxia, dystonia, and myoclonus, affecting the left arm and, to a lesser extent, the left leg more than the right side. She also had difficulty initiating saccadic eye movements. Repeat electromyography of the four limbs was normal. A diagnosis of CBS was made.

She continued to progress over the next few years with worsening comprehension of speech and increasing difficulty with movement. By 8 yr into the illness, she became dependent on nursing care for all her personal care needs and was not assessed further following this. Although not formally assessed, the cognitive impairment at this stage was of sufficient severity to significantly interfere with activities of daily living and contributed to her loss of independence. She died from bronchopneumonia at the age of 71, after a progressive neurodegenerative illness lasting 12 yr in duration.

**Figure 3.**
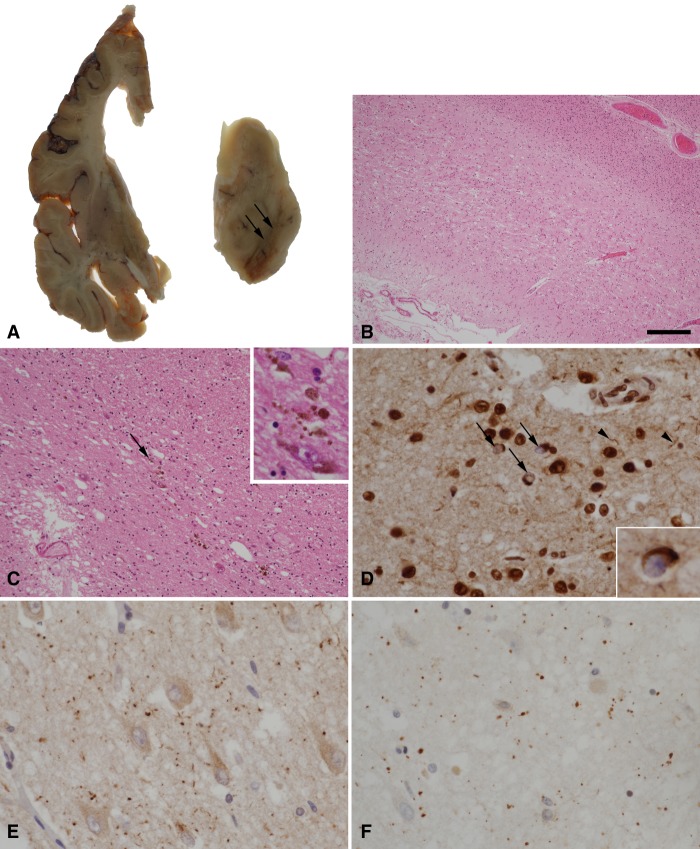
(*A*) A coronal slice from the left cerebral hemisphere demonstrates ventricular dilatation with atrophy of the frontal lobe, caudate nucleus, and amygdala. In the midbrain, there is severe pallor of the substantia nigra (arrows). (*B*) Histological examination of the frontal lobe by hematoxylin and eosin staining shows thinning and spongiosis of the cortex. (*C*) Severe loss of pigmented neurons in the substantia nigra is confirmed with residual free neuromelanin in the neuropil (arrow and *inset*). (*D*) TDP-43 immunohistochemistry in the frontal lobe shows neurons with loss of the normal nuclear staining pattern and containing cytoplasmic inclusions (arrows and *inset*), in addition to scattered short neurites (arrowheads). (*E,F*) Argyrophilic grains in the subiculum are highlighted by immunohistochemistry for p62 (*E*) and four-repeat tau isoforms (*F*). Scale bar, 300 µm (*B*), 120 µm (*C*), 30 µm (*C inset*, *D*,*E*,*F*), and 20 µm (*D inset*).

The patient consented to brain donation, and after death her brain was assessed using standard neuropathological methods at the Queen Square Brain Bank, where tissue is stored for research under a licence from the UK Human Tissue Authority. Representative areas of neocortex, basal ganglia, hippocampus, midbrain, pons, medulla, and cerebellum were examined using routine stains, with additional immunohistochemical staining for Aβ, tau (AT8), TDP-43, ubiquitin, p62, GFAP, CD68, and α-synuclein. Macroscopic examination showed marked atrophy of the frontal and parietal lobes and, to a lesser extent, atrophy of the temporal lobe with severe dilatation of the lateral ventricle. There was severe atrophy of the amygdala with moderate atrophy of the hippocampus. The caudate and globus pallidus were also atrophic and there was marked pallor of the substantia nigra and locus coeruleus (Fig. 3). Histological examination of the left hemisphere showed marked cortical pathology with thinning, neuronal loss, and spongiosis, most severe in the frontal and parietal lobes. There was neuronal loss and gliosis in the caudate and globus pallidus, and this was severe in the substantia nigra. TDP-43 immunohistochemistry demonstrated short neurites and neuronal cytoplasmic inclusions corresponding to cortical pathology characteristic in the FTLD–TDP type A subtype. Immunohistochemical staining for hyperphosphorylated tau showed features of argyrophilic grains with neurofibrillary tangles in the hippocampus accompanied by grains in the hippocampal CA1 subregion and the subiculum, in addition to small numbers of bushy astrocytes in cortical regions. There was no Aβ or α-synuclein pathology. The right hemisphere was not available for immunohistochemistry, as it was snap-frozen for molecular analysis.

### Genomic Analysis

Genetic screening excluded mutations in *GRN* and *MAPT* (exons 1 and 9–13). Hexanucleotide repeat expansions in the *C9orf72* gene, a cause of familial FTD and/or ALS (DeJesus-Hernandez et al. 2011; Renton et al. 2011), were also excluded. Exome sequencing was undertaken to identify potential pathogenic variants. The patient was found to harbor a novel c.2107G>T (p.E703X: NM_013254.4) variant in exon 20 of the *TBK1* gene (Table 1; Supplemental Table S1). Sanger sequencing of exon 20 confirmed the presence of a heterozygous G>T substitution at nucleotide 2107 (Fig. 2B). This variant was not seen in more than 138,000 unrelated exome and genome sequences from the gnomAD database (0/138,632; http://gnomad.broadinstititue.org/). This nonsense variant was located in the carboxy-terminal coiled-coil domain 2 (CC2) and results in a premature stop codon, leading to a predicted truncated protein product. Segregation of the variant could not be verified because of the unavailability of blood samples from additional family members.

**Table 1.** Details of variant

Gene	Chromosome	HGVS DNA reference	HGVS protein reference	Variant type	Predicted effect	dbSNP/dbVar ID	ClinVar ID	Genotype
*TBK1*	12q14.2	NM_013254.4: c.2107G>T	NP_037386.1: p.E703X	Nonsense variant	Premature STOP codon	Not available	SCV000886403	Heterozygous

## DISCUSSION

Here, we describe the presence of a novel *TBK1* mutation in a patient who presented clinically with a CBS–PNFA overlap syndrome, radiologically with asymmetric frontal lobe atrophy, and with FTLD–TDP type A pathology at postmortem examination of brain tissue.

Although we did not have access to blood samples to test segregation of the mutation within the family, this variant is highly likely to be pathogenic, as it results in a premature stop codon, and hence a truncated protein product. Heterozygous loss of function variants have been shown to be enriched in familial FTD–ALS pedigrees and are likely to be pathogenic through nonsense-mediated mRNA decay (NMD) and global reduction of protein levels (Cirulli et al. 2015; Freischmidt et al. 2015; Pottier et al. 2015). However, NMD associated with the heterozygous p.E703X mutation described here is unlikely, because of the position of the premature stop codon in the gene (Hug et al. 2016). Instead, loss of the crucial highly conserved CC2 protein domain is likely to result in the abrogation of the interaction and complex formation with TBK1-associated adaptors that are essential in regulating TBK1 activation and its subcellular localization (Gonçalves et al. 2011). Mutations within this domain have been shown to disrupt the TBK1/OPTN (optineurin) interaction (Freischmidt et al. 2015). OPTN is an important autophagy receptor critical for the degradation and clearance of intracellular pathogens, protein aggregates, and damaged organelles. The carboxy-terminal domain of TBK1 binds to the amino-terminal region of OPTN to form the TBK1/OPTN complex (Li et al. 2016). TBK1 is also thought to directly phosphorylate OPTN to promote autophagy. Mutations in both the carboxy-terminal domain of TBK1 and the amino-terminal domain of OPTN have been implicated in ALS and other neurodegenerative diseases (Maruyama et al. 2010; Pottier et al. 2015), and it is likely that disruption of the formation of the TBK1/OPTN complex impairs critical autophagy processes that are vital to maintaining cellular homeostasis (Li et al. 2016). Interestingly, TBK1 has also been shown to act on a common pathway with C9orf72 complexes to regulate autophagy in neuronal cells (Sellier et al. 2016). It was found that by promoting the phosphorylation of one of the proteins in this complex (SMCR8), TBK1 is important for C9orf72-mediated autophagy, thus providing another mechanism by which TBK1 mutations could lead to neurodegeneration. Disruption in autophagy processes has been implicated in other neurodegenerative conditions such as Parkinson's disease, in which mutations in *PINK1* and *Parkin* are believed to function in a common pathway to promote defective mitochondrial clearance via autophagy (Barodia et al. 2017).

Clinically, this patient developed an overlap syndrome of primary progressive aphasia (PNFA) followed by CBS. This is a well-described overlap of neurodegenerative syndromes, but only one other case has been described in association with a *TBK1* frameshift mutation (Caroppo et al. 2015). Neuroanatomically, there are few reports of the imaging features of *TBK1*-associated disease. Patterns of atrophy are variable and likely reflect the areas most affected by neurodegeneration. In most cases, atrophy is asymmetric and predominantly involves the temporal lobe (Caroppo et al. 2015; Koriath et al. 2016). However, atrophy that predominantly affects the frontal cortex, mesencephalon, and cerebellum have all been described, and it is plausible that atrophy patterns correlate with the main clinical phenotype (Wilke et al. 2018). The neuropathological features associated with *TBK1* mutations have also been described infrequently. Most cases reported so far had TDP-43 type B pathology (Freischmidt et al. 2015; Pottier et al. 2015; Van Mossevelde et al. 2016), but TDP-43 type A pathology has also been described (Pottier et al. 2015; Koriath et al. 2017). Whether there is a correlation between mutation type and/or location within the different TBK1 domains and the pathological consequences remains to be elucidated. Of interest, this case also had tau-positive argyrophilic grains, as previously reported, raising the possibility that this additional pathology might be a feature of the disease (Koriath et al. 2017).

Mutations in *TBK1* are the fourth most common cause of familial FTD, and the second most common cause of a combined FTD–ALS syndrome (Dols-Icardo et al. 2018). More recently, *TBK1* mutations have been found in patients with progressive supranuclear palsy and progressive cerebellar ataxia syndromes, expanding the phenotypic spectrum of *TBK1*-associated disease (Wilke et al. 2018). Only one other case of familial CBS–PNFA overlap syndrome has been reported in the literature thus far, carrying a frameshift mutation (p.Thr156ArgfsX6) affecting the kinase domain of TBK1 (Caroppo et al. 2015). The first described cause of familial CBS was due to a mutation in the *MAPT* gene (Masellis et al. 2006), but this is relatively rare (van Swieten 2007) and tends to cause a syndrome with prominent behavioral symptoms. A familial CBS overlap with primary progressive aphasia is more commonly associated with *GRN* mutations (van Swieten and Heutink 2008), but is nonetheless a rare phenotype of *GRN*. More recently, case reports of familial CBS caused by *C9orf72* and *CSF1R* have been described, but this is also rare and an unusual phenotype for these genes. It is increasingly recognized that neurodegenerative disorders are pathologically and genetically heterogeneous. Similar clinical syndromes can have multiple underlying pathologies and genetic cause, and mutations in a single gene can manifest with diverse clinical phenotypes, often in the same kindred. In this context, clearly exemplified by this case report, the exact definition of a clinicopathological entity is increasingly reliant on a genetic diagnosis.

In summary, we describe a novel *TBK1* mutation in a patient who presented with a CBS–PNFA overlap and who had a family history of dementia and ALS. The presence of a family history in a patient with CBS should lead to testing for mutations in the known FTD genes. However, if these are negative sequencing for mutations in *TBK1* should be considered. This case highlights the importance of genetic exome sequencing as a diagnostic tool in patients presenting with CBS and associated neurodegenerative disorders.

## METHODS

Briefly, for whole-exome sequencing, the sample library was prepared using Illumina Nextera Rapid Capture kits with paired-end sequencing performed using the Illumina HiSeq2000 (Illumina). The sample was sequenced to an average coverage of 39.42 reads with a mean read length of 177.23 bp (Supplemental Table S2). All reads were subsequently aligned using BWA (Li and Durbin 2009) against UCSC hg19 reference genome. Variant calling and quality-based filtering for all samples were done using GATK (McKenna et al. 2010). Variants were annotated with ANNOVAR (Wang et al. 2010) with allele frequency data from the gnomAD browser, as well as with predicted impact of variants (Kircher et al. 2014).

For Sanger sequencing, exon 20 was amplified using specific primers (F 5′-CAGCTTCCAGTGGAATCAAACA-3′ and R 5′-AGGCATCACAGATACACAATCA-3′). The amplified PCR product was enzymatically cleaned up and sequenced on both strands using a BigDye Terminator v3.1 cycle sequencing kit (Applied Biosystems). Sequencing products were run on a 3730 DNA Analyser (Applied Biosystems) and analyzed using Sequencher DNA Sequence Analysis software (Gene Codes Corp.).

## ADDITIONAL INFORMATION

### Data Deposition and Access

Raw sequencing data were not deposited but are available from the authors. The variant described in this study was submitted to ClinVar (http://www.ncbi.nlm.nih.gov/clinvar/) and can be found under accession number SCV000886403.

### Ethics Statement

The patient and her next of kin gave written consent to take part in research. Genetic and postmortem neuropathological analysis of this patient was approved under the Research Ethics Committee for Wales (Wales REC; 14/WA/1179; Clinical Neurological Disease Bio-bank and Neurogenetics Research Study 2 (CANDAS2)).

### Acknowledgments

We thank Dr. Viorica Chelban for her assistance with the Sanger sequencing. This research was partly supported by the National Institute for Health Research (NIHR) Queen Square Biomedical Research Unit in Dementia based at University College London Hospitals (UCLH), University College London (UCL). The views expressed are those of the authors and not necessarily those of the National Health Service (NHS), the National Institute for Health Research (NIHR), or the Department of Health.

### Author Contributions

The project was conceived and supervised by H.R.M. and D.J.B. R.L., J.D.R., A.J.W., S.J.L., T.L., T.R., J.L.H., R.J.W., H.R.M., and R.R. generated and analyzed data. J.D.R., R.R., and R.L. wrote the manuscript. All authors were responsible for critical review and interpretation of the data.

### Funding

R.L. is funded by CBD Solutions. The Dementia Research Centre is supported by Alzheimer's Research UK, Brain Research Trust, and The Wolfson Foundation. This work was supported by the NIHR Queen Square Dementia Biomedical Research Unit, the NIHR UCL/H Biomedical Research Centre, and the Leonard Wolfson Experimental Neurology Centre (LWENC) Clinical Research Facility. J.D.R. is supported by an MRC Clinician Scientist Fellowship (MR/M008525/1) and has received funding from the NIHR Rare Disease Translational Research Collaboration (BRC149/NS/MH). A.J.W. is supported by BRACE. T.L. is supported by an Alzheimer's Research UK senior fellowship. T.R. is supported by a research grant from Karin & Sten Mortstedt CBD Solutions. J.L.H. is supported by the Multiple System Atrophy Trust, the Multiple System Atrophy Coalition, Fund Sophia, managed by the King Baudouin Foundation, Alzheimer's Research UK, and CBD Solutions. R.R. is a Walker-Peltz Clinical Research Fellow. S.J.L. was funded by the Medical Research Council UK (G0700943). Queen Square Brain Bank is supported by the Reta Lila Weston Institute of Neurological Studies and the Medical Research Council UK.

### Competing Interest Statement

The authors have declared no competing interest.

### Referees

Pamela McCombe

Anonymous

## Supplementary Material

Supplemental Material

## References

[MCS003913LAMC1] Ahmad L, Zhang S, Casanova J, Sancho-Shimizu V. 2016 Human *TBK1*: a gatekeeper of neuroinflammation. Trends Mol Med 22: 511–527. 10.1016/j.molmed.2016.04.006 27211305 PMC4890605

[MCS003913LAMC2] Anor CJ, Xi Z, Zhang M, Moreno D, Sato C, Rogaeva E, Tartaglia MC. 2015 Mutation analysis of *C9orf72* in patients with corticobasal syndrome. Neurobiol Aging 36: 2905.e1–2905.e5. 10.1016/j.neurobiolaging.2015.06.008 26166205

[MCS003913LAMC3] Armstrong MJ, Litvan I, Lang AE, Bak TH, Bhatia KP, Borroni B, Boxer AL, Dickson DW, Grossman M, Hallett M, 2013 Criteria for the diagnosis of corticobasal degeneration. Neurology 80: 496–503. 10.1212/WNL.0b013e31827f0fd1 23359374 PMC3590050

[MCS003913LAMC4] Barodia SK, Creed RB, Goldberg MS. 2017 *Parkin* and *PINK1* functions in oxidative stress and neurodegeneration. Brain Res Bull 133: 51–59. 10.1016/j.brainresbull.2016.12.004 28017782 PMC5718625

[MCS003913LAMC5] Caroppo P, Camuzat A, De Septenville A, Couratier P, Lacomblez L, Auriacombe S, Flabeau O, Jornéa L, Blanc F, Sellal F, 2015 Semantic and nonfluent aphasic variants, secondarily associated with amyotrophic lateral sclerosis, are predominant frontotemporal lobar degeneration phenotypes in *TBK1* carriers. Alzheimers Dement 1: 481–486. 10.1016/j.dadm.2015.10.002 PMC4879495 27239526

[MCS003913LAMC6] Cirulli ET, Lasseigne BN, Petrovski S, Sapp PC, Dion PA, Leblond CS, Couthouis J, Lu YF, Wang Q, Krueger BJ, 2015 Exome sequencing in amyotrophic lateral sclerosis identifies risk genes and pathways. Science 347: 1436–1441. 10.1126/science.aaa3650 25700176 PMC4437632

[MCS003913LAMC7] DeJesus-Hernandez M, Mackenzie IR, Boeve BF, Boxer AL, Baker M, Rutherford NJ, Nicholson AM, Finch NA, Flynn H, Adamson J, 2011 Expanded GGGGCC hexanucleotide repeat in noncoding region of *C9ORF72* causes chromosome 9p-linked FTD and ALS. Neuron 72: 245–256. 10.1016/j.neuron.2011.09.011 21944778 PMC3202986

[MCS003913LAMC8] Dols-Icardo O, García-Redondo A, Rojas-García R, Borrego-Hernández D, Illán-Gala I, Muñoz-Blanco JL, Rabano A, Cervera-Carles L, Juárez-Rufián A, Spataro N, 2018 Analysis of known amyotrophic lateral sclerosis and frontotemporal dementia genes reveals a substantial genetic burden in patients manifesting both diseases not carrying the *C9orf72* expansion mutation. J Neurol Neurosurg Psychiatry 89: 162–168. 10.1136/jnnp-2017-316820 28889094

[MCS003913LAMC9] Freischmidt A, Wieland T, Richter B, Ruf W, Schaeffer V, Müller K, Marroquin N, Nordin F, Hübers A, Weydt P, 2015 Haploinsufficiency of *TBK1* causes familial ALS and fronto-temporal dementia. Nat Neurosci 18: 631–636. 10.1038/nn.4000 25803835

[MCS003913LAMC10] Freischmidt A, Müller K, Ludolph AC, Weishaupt JH, Andersen PM. 2017 Association of mutations in *TBK1* with sporadic and familial amyotrophic lateral sclerosis and frontotemporal dementia. JAMA Neurol 74: 110–113. 10.1001/jamaneurol.2016.3712 27892983

[MCS003913LAMC11] Gonçalves A, Bürckstümmer T, Dixit E, Scheicher R, Górna MW, Karayel E, Sugar K, Stukalov A, Berg T, Kralovics R, 2011 Functional dissection of the *TBK1* molecular network. PLoS One 6: e23971 10.1371/journal.pone.0023971 21931631 PMC3169550

[MCS003913LAMC12] Hug N, Longman D, Cáceres JF. 2016 Mechanism and regulation of the nonsense-mediated decay pathway. Nucleic Acids Res 44: 1483–1495. 10.1093/nar/gkw010 26773057 PMC4770240

[MCS003913LAMC13] Kircher M, Witten DM, Jain P, O'Roak BJ, Cooper GM, Shendure J. 2014 A general framework for estimating the relative pathogenicity of human genetic variants. Nat Genet 46: 310–315. 10.1038/ng.2892 24487276 PMC3992975

[MCS003913LAMC14] Koriath C, Adamson G, Druyeh R, Kenny J, Rossor M, Schott J, Collinge J, Fox N, Roher J, Mead S. 2016 Probing FTD genetics with next-generation sequencing. J Neurol Neurosurg Psychiatry 87: e1.198-e1 10.1136/jnnp-2016-315106.61

[MCS003913LAMC15] Koriath CA, Bocchetta M, Brotherhood E, Woollacott IOC, Norsworthy P, Simón-Sánchez J, Blauwendraat C, Dick KM, Gordon E, Harding SR, 2017 The clinical, neuroanatomical, and neuropathologic phenotype of *TBK1*-associated frontotemporal dementia: a longitudinal case report. Alzheimers Dement 6: 75–81. 10.1016/j.dadm.2016.10.003 PMC5312484 28229125

[MCS003913LAMC16] Li H, Durbin R. 2009 Fast and accurate short read alignment with Burrows–Wheeler transform. Bioinformatics 25: 1754–1760. 10.1093/bioinformatics/btp324 19451168 PMC2705234

[MCS003913LAMC17] Li F, Xie X, Wang Y, Liu J, Cheng X, Guo Y, Gong Y, Hu S, Pan L. 2016 Structural insights into the interaction and disease mechanism of neurodegenerative disease-associated optineurin and TBK1 proteins. Nat Commun 7: 12708 10.1038/ncomms12708 27620379 PMC5027247

[MCS003913LAMC18] Ling H, O'Sullivan SS, Holton JL, Revesz T, Massey LA, Williams DR, Paviour DC, Lees AJ. 2010 Does corticobasal degeneration exist? A clinicopathological re-evaluation. Brain 133: 2045–2057. 10.1093/brain/awq123 20584946

[MCS003913LAMC19] Marion J. 2014 TANK-binding kinase 1 (TBK1): structure, function, and regulation. In Molecular life sciences: an encyclopedic reference (Wells RD, Bond JS, Klinman J, Masters BSS, Bell E), pp. 1–9. Springer, New York.

[MCS003913LAMC20] Maruyama H, Morino H, Ito H, Izumi Y, Kato H, Watanabe Y, Kinoshita Y, Kamada M, Nodera H, Suzuki H, 2010 Mutations of optineurin in amyotrophic lateral sclerosis. Nature 465: 223–226. 10.1038/nature08971 20428114

[MCS003913LAMC21] Masellis M, Momeni P, Meschino W, Heffner R, Elder J, Sato C, Liang Y, St George-Hyslop P, Hardy J, Bilbao J, 2006 Novel splicing mutation in the progranulin gene causing familial corticobasal syndrome. Brain 129: 3115–3123. 10.1093/brain/awl276 17030534

[MCS003913LAMC22] McKenna A, Hanna M, Banks E, Sivachenko A, Cibulskis K, Kernytsky A, Garimella K, Altshuler D, Gabriel S, Daly M, 2010 The Genome Analysis Toolkit: a MapReduce framework for analyzing next-generation DNA sequencing data. Genome Res 20: 1297–1303. 10.1101/gr.107524.110 20644199 PMC2928508

[MCS003913LAMC23] Pottier C, Bieniek KF, Finch N, van de Vorst M, Baker M, Perkersen R, Brown P, Ravenscroft T, van Blitterswijk M, Nicholson AM, 2015 Whole-genome sequencing reveals important role for *TBK1* and *OPTN* mutations in frontotemporal lobar degeneration without motor neuron disease. Acta Neuropathol 130: 77–92. 10.1007/s00401-015-1436-x 25943890 PMC4470809

[MCS003913LAMC24] Rademakers R, Baker M, Nicholson AM, Rutherford NJ, Finch N, Soto-Ortolaza A, Lash J, Wider C, Wojtas A, DeJesus-Hernandez M, 2012 Mutations in the colony stimulating factor 1 receptor (*CSF1R*) gene cause hereditary diffuse leukoencephalopathy with spheroids. Nat Genet 44: 200–205. 10.1038/ng.1027 PMC3267847 22197934

[MCS003913LAMC25] Renton AE, Majounie E, Waite A, Simón-Sánchez J, Rollinson S, Gibbs JR, Schymick JC, Laaksovirta H, van Switen JC, Myllykangas L, 2011 A hexanucleotide repeat expansion in *C9ORF72* is the cause of chromosome 9p21-linked ALS-FTD. Neuron 72: 257–268. 10.1016/j.neuron.2011.09.010 21944779 PMC3200438

[MCS003913LAMC26] Sellier C, Campanari ML, Corbier CJ, Gaucherot A, Kolb-Cheynel I, Oulad-Abdelghani M, Ruffenach F, Page A, Ciura S, Kabashi E, 2016 Loss of C9ORF72 impairs autophagy and synergizes with polyQ ataxin-2 to induce motor neuron dysfunction and cell death. EMBO J 35: 1276–1297. 10.15252/embj.201593350 27103069 PMC4910533

[MCS003913LAMC27] Van Mossevelde S, van der Zee J, Gijselinck I, Engelborghs S, Sieben A, Van Langenhove T, De Bleecker J, Baets J, Vandenbulcke M, Van Laere K, 2016 Clinical features of *TBK1* carriers compared with *C9orf72*, *GRN* and non-mutation carriers in a Belgian cohort. Brain 139: 452–467. 10.1093/brain/awv358 26674655 PMC4805085

[MCS003913LAMC28] van Swieten JC. 2007 Genetic basis of frontotemporal dementia. Lancet Neurol 6: 840–841. 10.1016/S1474-4422(07)70224-7 17884666

[MCS003913LAMC29] van Swieten JC, Heutink P. 2008 Mutations in progranulin (GRN) within the spectrum of clinical and pathological phenotypes of frontotemporal dementia. Lancet Neurol 7: 965–974. 10.1016/S1474-4422(08)70194-7 18771956

[MCS003913LAMC30] Wang K, Li M, Hakonarson H. 2010 ANNOVAR: functional annotation of genetic variants from high-throughput sequencing data. Nucleic Acids Res 38: e164 10.1093/nar/gkq603 20601685 PMC2938201

[MCS003913LAMC31] Wilke C, Baets J, De Bleecker JL, Deconinck T, Biskup S, Hayer SN, Hayer SN, Züchner S, Schüle R, De Jonghe P, 2018 Beyond ALS and FTD: the phenotypic spectrum of *TBK1* mutations includes PSP-like and cerebellar phenotypes. Neurobiol Aging 62: 244.e9–244.e13. 10.1016/j.neurobiolaging.2017.10.010 29137817

