# Supplementary material for: A novel TBK1 mutation in a family with diverse frontotemporal dementia spectrum disorders
Source: Cold Spring Harb Mol Case Stud. 2019 Jun;5(3):a003913. doi: 10.1101/mcs.a003913 (PMC6549548; doi:10.1101/mcs.a003913)
Supplement: Supplemental Material [file supp_5_3_a003913__index.html]

Supplemental Material 

# A novel *TBK1* mutation in a family with diverse frontotemporal dementia spectrum disorders

## Supplemental Material

- Supplemental\_Movie\_S1.mov
- Supplemental\_Table\_S1\_Variant\_interpretation.xlsx
- Supplemental\_Table\_S2\_Whole-exome\_sequencing\_parameters.xlsx
